# Functional Connectivity Modulation by Acupuncture in Patients with Bell's Palsy

**DOI:** 10.1155/2016/5928758

**Published:** 2016-05-12

**Authors:** Yunpeng Bian, Xiaoxuan He, Sheng Hu, Chuanfu Li, Chunsheng Xu, Hongxing Kan, Qiuju Xue, Jun Yang, Bensheng Qiu

**Affiliations:** ^1^Center for Biomedical Engineering, University of Science and Technology of China, Hefei, Anhui 230027, China; ^2^Laboratory of Digital Medical Imaging, Medical Imaging Center, The First Affiliated Hospital, Anhui University of Chinese Medicine, Hefei, Anhui 230031, China; ^3^Anhui Computer Application Institute of Traditional Chinese Medicine, Hefei, Anhui 230038, China; ^4^Department of Radiology, University of Washington School of Medicine, Seattle, WA 98108, USA

## Abstract

Bell's palsy (BP), an acute unilateral facial paralysis, is frequently treated with acupuncture in many countries. However, the mechanism of treatment is not clear so far. In order to explore the potential mechanism, 22 healthy volunteers and 17 BP patients with different clinical duration were recruited. The resting-state functional magnetic resonance imaging scans were conducted before and after acupuncture at LI4 (Hegu), respectively. By comparing BP-induced functional connectivity (FC) changes with acupuncture-induced FC changes in the patients, the abnormal increased FC that could be reduced by acupuncture was selected. The FC strength of the selected FC at various stages was analyzed subsequently. Our results show that FC modulation of acupuncture is specific and consistent with the tendency of recovery. Therefore, we propose that FC modulation by acupuncture may be beneficial to recovery from the disease.

## 1. Introduction

Bell's palsy (BP), an acute unilateral facial paralysis, is a frequent facial movement dysfunction caused by the impaired facial nerve that controls movement of facial muscles [1–3]. Acupuncture, a traditional Chinese medicine [4, 5], has been considered to have a beneficial effect on the acute state of BP in the last few years [6]. Although acupuncture is used in treatment of BP in East Asia, the mechanism has not been completely elucidated so far [7].

Functional magnetic resonance imaging (fMRI), a noninvasive brain imaging technique, is widely used in the assessment of the effect of acupuncture [8, 9]. There are many fMRI investigations on how acupuncture affects healthy people, suggesting that acupuncture can adjust brain functional network [10–13]. In the meantime, more and more neuroimaging studies showed that acupuncture had the same effect on the brain functional network in patients with certain diseases [14]. For instance, acupuncture enhanced the hippocampal connectivity in patients with Alzheimer [15, 16]. Acupuncture also modulated endogenous pain regulation network, valued by analgesic effects in low-back and leg pain patients [17].

Our previous study has shown that functional connectivity (FC) in primary somatosensory area is modulated by acupuncture in BP patients [18]. Concomitantly, several studies on BP used fMRI to explore neuroplasticity, demonstrating that the altered FC occurred in the cortical facial motor network at early stage of the disease and gradually recovered during subsequent cortical reorganization [19–23]. However, it is unclear whether acupuncture could have any effect on the aforementioned altered FC in patients with BP, and whether such modulation would be beneficial to recovery. If these problems can be solved, it will be helpful to reveal the underlying mechanism of acupuncture treatment.

To explore the questions above, the regions of interest (ROIs) were firstly extracted from the activation mapping of BP patients undertaking acupuncture and the facial motor related regions. The intergroup analysis was conducted between healthy control and patients before acupuncture to extract the altered FC at the onset time. The intergroup analysis of post- and preacupuncture BP patients was then conducted to find out FC changes modulated by acupuncture. Thereafter, the overlapping regions in the two mappings were generated. FC intensity at various stages was also calculated to compare the tendency of recovery with modulation by acupuncture.

## 2. Materials and Methods

### 2.1. Subjects

This study was approved by the institutional review board of the First Affiliated Hospital of Anhui University of Chinese Medicine, and signed written informed consent was collected before the study. A total of 17 BP patients and 22 healthy volunteers were recruited, who were right handed and confirmed having no history of stroke, severe hypertension, drug abuse, psychiatric diseases, systematic diseases, or other serious diseases that might affect the study results. Both the healthy volunteers and the BP patients were acupuncture naïve before participating in the experiment. In the healthy group, all volunteers (13 males and 9 females, mean age 25.3 ± 3.4 years) were either students or staffs from the First Affiliated Hospital of Anhui University of Chinese Medicine. All BP patients (11 males and 6 females, mean age 33.5 ± 12.0 years) had right unilateral facial paresis and received acupuncture treatment three times a week till recovery. On the basis of House-Brackmann facial paresis (HBS scoring system of facial movement: 1 = normal facial movement, 6 = no movement [24]) and onset duration, all these patients were ranked as HBS ≥ III at the onset time of the disease and HBS = I when they got recovered. While healthy individuals undertook MRI examination only once, BP patients undertook the scanning at the onset time and after the recovery (due to loss of contact, one female did not receive scanning after the recovery), with the data used as the patient group and recovered group, respectively.

### 2.2. Data Acquisition of Acupuncture fMRI

The data acquisition was performed on a 1.5 T MRI whole body scanner (Symphony, Siemens Healthcare, Germany) with a standard head coil at the First Affiliated Hospital of Anhui University of Chinese Medicine. The experiment took about 60 minutes and a total of eight imaging sequences were scanned: (a) pilot images; (b) T2-weighted images, which could exclude any obvious diseases of the brain; (c) T1-weighted 2D anatomical images (SE sequence with TR/TE = 500/12 ms, field of view (FOV) = 230 × 230 mm, slice thickness/interval = 3.0/0.75 mm, resolution = 192 × 144, and a total of 36 slices) that covered the whole brain and paralleled to the anterior commissure-posterior commissure line; (d) resting-state fMRI images obtained before acupuncture using an echo-planar imaging (EPI) sequence (TR/TE/FA = 3000 ms/30 ms/90°); (e) resting-state fMRI images during acupuncture acquired with the same parameters; (f) resting-state fMRI images after acupuncture acquired with the same parameters; (g) task-state fMRI images acquired using EPI sequence (TR/TE/FA = 4000 ms/50 ms/90°); (h) T1-weighted 3D anatomical images, a total of 176 slices that covered the whole brain, using a spoiled gradient echo sequence (TR/TE/FA = 2100 ms/3.93 ms/13°, FOV = 250 mm × 250 mm, slice thickness/interval = 1.0/0.5 mm, and resolution = 256 × 256). All participants were trained to relax before the scanning and close their eyes, keep silent, and avoid psychological activity during the scanning. All the lights in the scanning room were turned off to reduce visual stimulation.

### 2.3. Functional MRI Paradigms

A 10-minute resting-state data was first acquired before acupuncture. Then, a stainless steel acupuncture needle was inserted into Hegu on the dorsum of left hand at 1 cm skin depth. LI4 or Hegu, located on the dorsum of the hand, is an important commonly used acupuncture point. When a de-qi sensation was obtained, the resting-state data was beginning to be acquired during acupuncture. This scan also lasted 10 minutes, and the needle was retained and rotated for 10 seconds every 2 minutes. Finally, the needle was pulled out and the third resting-state data was then acquired in the next 10 minutes.

Task-state data lasted 10 minutes and 40 seconds during acupuncture. There were 160 volumes (with TR 4 seconds) in this acquisition. As with the resting-state data acquisition, the needle was retained in the skin until de-qi before the acquisition began. In this paradigm, the needle retaining and twirling were conducted alternately, and the needle was rotated bidirectionally with an even motion at the rate of 1 Hz. The entire fMRI acquisition scheme was as follows: 32 volumes of retaining, 32 volumes of twirling, 48 volumes of retaining, 32 volumes of twirling, and finally 16 volumes of retaining (see Figure S2 in Supplementary Material available online at http://dx.doi.org/10.1155/2016/5928758).

### 2.4. Imaging Preprocessing

The imaging preprocessing as well as data analysis was performed using analysis of functional neuroimages (AFNI) software (http://afni.nimh.nih.gov/, Medical College of Wisconsin, Milwaukee, Wisconsin, USA). Considering the magnetic equilibrium, the first four images of each functional session were discarded. Thereafter, all the functional and anatomical images were preprocessed (reconstructed, corrected for slice acquisition time, corrected for motion, and skull stripped and smoothed with a Gaussian kernel of 6-mm full-width at half maximum). The head movements of all the data were less than 2 mm or 2°. All fMRI data was then filtered (0.007–0.1 Hz) to remove low-frequency drift and high-frequency noises. Then, the functional images were coregistered to the anatomical images and normalized to the MNI152 standard brain. All the normalized images were resliced by 3.0 × 3.0 × 3.0 mm^3^ voxels. After preprocessing, the individual transformed 4D data was used for further analysis.

### 2.5. Extraction of the Region of Interest

The task-state fMRI data of the patient group was analyzed to produce the statistical activation map. The results of group analysis were corrected with Monte Carlo method, in which the threshold of statistical significance was set to *p* = 0.01, *α* = 0.05, and a minimum cluster size of 38 voxels (3dClustSim, AFNI). On the basis of the map, we extracted all the “peaks” of the activation areas as seed regions (Table S1).

In order to verify whether acupuncture could regulate the motor network in BP patients, we selected the motor areas activated by BP patient's mouth movement task. We selected 10 seed regions in the activation map of the nonaffected side motor task by Klingner's test [22] (Table S2). All the seed regions were extracted manually, with a diameter of 4 mm, containing 10 voxels (voxel size 3.0 × 3.0 × 3.0 mm^3^).

### 2.6. Functional Connectivity Analyses

FC of each seed region was computed, respectively. First, the temporal signal series of white matter (WM), cerebrospinal fluid (CSF), and seed regions were extracted. Then, a linear regression analysis was conducted to remove the following confounding sources: (a) six motion parameters, (b) linear drift, (c) white matter signal, and (d) CSF signal. Thereafter, the individual statistical maps were obtained based on the general linear model for further group analysis.

### 2.7. Functional Connectivity Strength Analyses

We extracted the regions with abnormal FC where acupuncture modulated FC changes could also be observed in BP patients. In order to calculate the selected FC strength for all groups, we first obtained the mean time series of each of the selected regions (Figure S1, Table 1) by averaging the fMRI time series over all voxels in the region. Then, the Pearson correlation coefficients were computed between all pairs of the selected regions for each subject in all groups. In the end, Fisher's *r*-to-*z* transformation was applied to correlation coefficients in order to increase normality of the distribution.

### 2.8. Group Analysis

All the intergroup comparisons were performed through paired *t*-test between post- and preacupuncture statistical maps. All the intergroup comparisons of different groups before acupuncture were performed through two-sample *t*-tests. Monte Carlo correction was done to control the false discovery rate. The results of intergroup analysis were corrected with *p* = 0.05, *α* = 0.05, and a minimum cluster size of 155 voxels (3dClustSim, AFNI). Two-sample *t*-tests were performed to compare the connectivity strength values in the intergroup analysis.

## 3. Results

**Figure 1:**
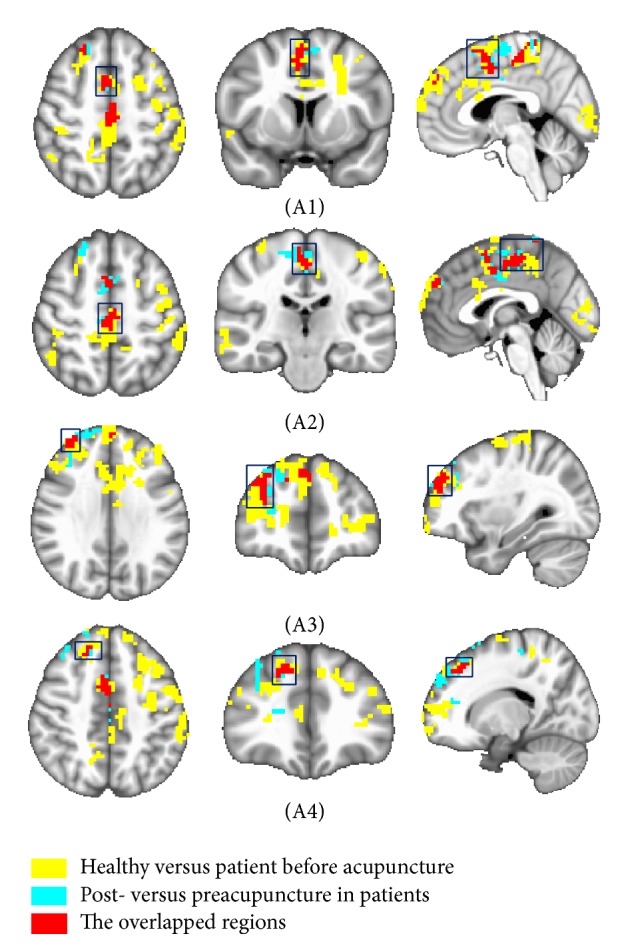
Demonstration of the overlapped regions which were selected from two intergroup comparison functional connectivity (FC) results of left middle frontal gyrus. The red areas represented the overlapped areas, which were detected from comparing BP-induced FC changes with acupuncture-induced FC changes in the patients. The overlapped areas were found in the bilateral supplementary motor area (A1, A2) and right superior frontal gyrus (A3, A4). The yellow areas represented the intergroup comparison FC results of areas between the healthy group and the patient group before acupuncture. The blue areas represented the intergroup comparison FC results of areas between post- and preacupuncture in the patient group. The threshold was set to *p* ≤ 0.05, *α* ≤ 0.05, corrected using the Monte Carlo method.

Firstly, 17 ROIs were selected from acupuncture needling response (Table S1) and BP patients' motor task (Table S2), and the FC of each ROI in these cases was analyzed. Here, the seed point left middle frontal gyrus (MFG) was used as an example since all the FC analysis procedures for each ROI were the same. To investigate BP-induced changes in FC of left MFG, the preacupuncture FC of the healthy group was compared with that of the patient group. The significantly negative regions (blue areas, Figure S3A, Table S3) were found in the results that demonstrate the abnormal increased FC in the patient group. In order to explore acupuncture-induced changes in FC of left MFG in BP patients, we also compared FC between post- and preacupuncture in the patient group and found that acupuncture could reduce FC in patients with BP (Figure S3B, Table S4). We next set out to investigate whether acupuncture could reduce the abnormal increased FC in patients. The above-mentioned two intergroup results of left MFG were compared, and the overlapped areas were obtained in the bilateral supplementary motor area (SMA) and right superior frontal gyrus (SFG) of these results (Figure S3C). The overlapped regions were selected and demonstrated the abnormal increased FC that could be reduced by acupuncture in the patient group (Figure 1).

**Figure 2 fig2:**
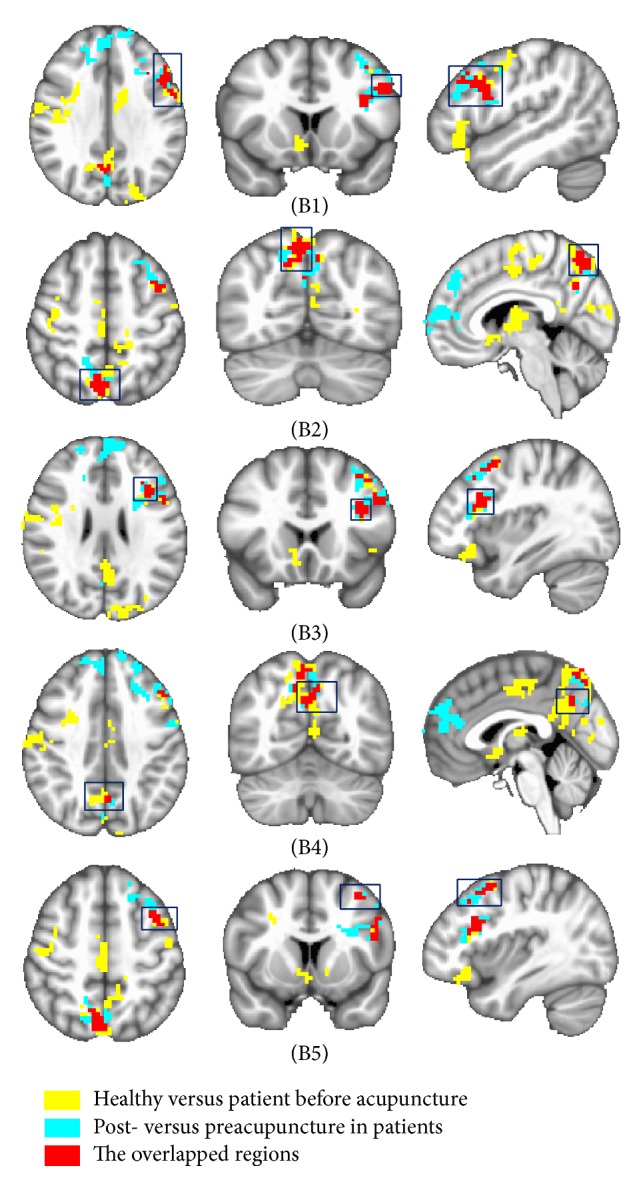
Demonstration of the overlapped regions which were selected from two intergroup comparison functional connectivity (FC) results of left primary motor cortex. The red areas represented the overlapped areas, which were detected from comparing BP-induced FC changes with acupuncture-induced FC changes in the patients. The overlapped areas were found in the left middle frontal gyrus (B1, B3, B5), left precuneus (B4), and right precuneus (B2). The yellow areas represented the intergroup comparison FC results of areas between the healthy group and the patient group before acupuncture. The blue areas represented the intergroup comparison FC results of areas between post- and preacupuncture in the patient group. The threshold was set to *p* ≤ 0.05, *α* ≤ 0.05, corrected using the Monte Carlo method.

**Figure 3:**
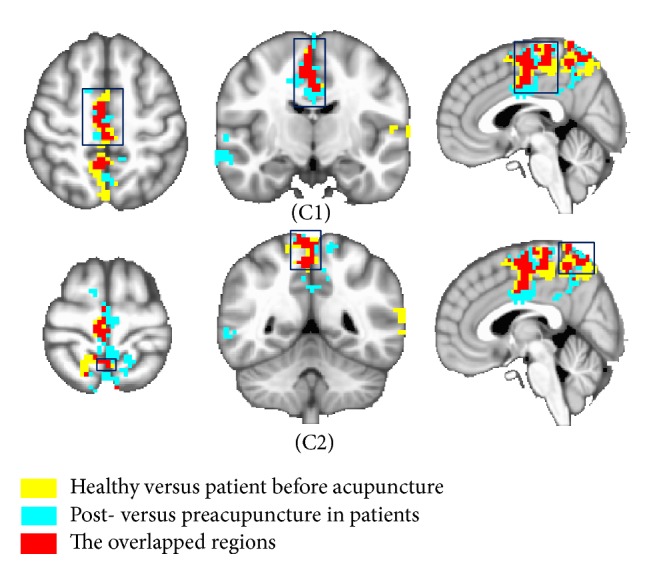
Demonstration of the overlapped regions which were selected from two intergroup comparison functional connectivity (FC) results of right cingulate motor area. The red areas represented the overlapped areas, which were detected from comparing BP-induced FC changes with acupuncture-induced FC changes in the patients. The overlapped areas were found in the right supplementary motor area (C1) and right primary somatosensory cortex (C2). The yellow areas represented the intergroup comparison FC results of areas between the healthy group and the patient group before acupuncture. The blue areas represented the intergroup comparison FC results of areas between post- and preacupuncture in the patient group. The threshold was set to *p* ≤ 0.05, *α* ≤ 0.05, corrected using the Monte Carlo method.

The same analysis method described above was applied to other selected seed points, respectively. Similarly, we compared those intergroup results of left primary motor cortex (MI) and determined overlapped areas in bilateral precuneus and left MFG (Figures 2 and S4). In right cingulate motor area (CMA), acupuncture could reduce abnormal FC in right SMA and primary somatosensory cortex (SI) (Figures 3 and S5). No other overlapped regions were found in comparison of these intergroup analysis results about the other remaining ROIs.

**Figure 4:**
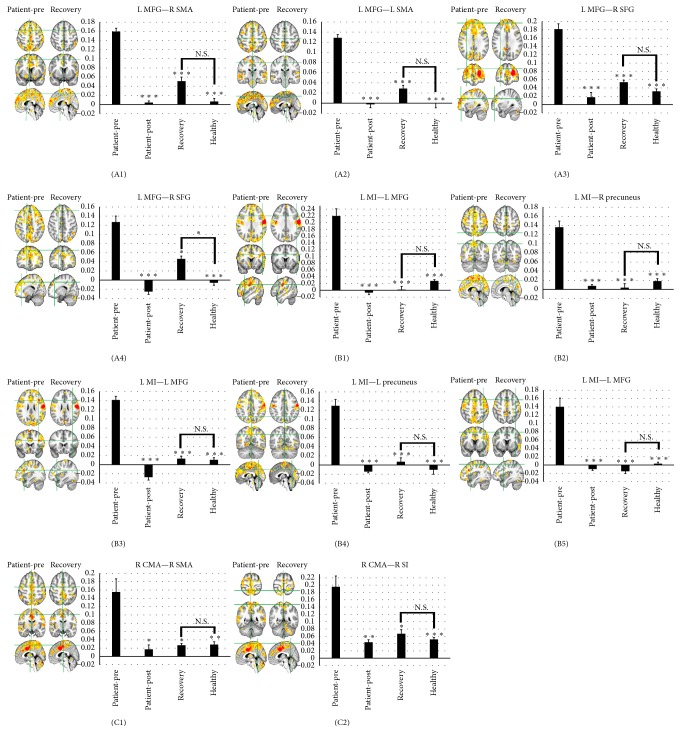
The histogram of functional connectivity strength and the resting fMRI maps of the selected ROIs and their overlapped regions for all groups. We found that the trend of functional connectivity changes induced by acupuncture was consistent with the changes induced by rehabilitation. Reduction of this functional connectivity was observed after acupuncture as well as during recovery process. The *x*-axis represents different groups and *y*-axis represents the functional connectivity strength. “Patient-pre” represents the patient group before acupuncture, “patient-post” represents the patient group after acupuncture, “recovery” represents the recovered group before acupuncture, and “healthy” represents the healthy group before acupuncture. Mean ± SEM, ^*∗∗∗*^ *p* < 0.005, ^*∗∗*^ *p* < 0.01, and ^*∗*^ *p* < 0.05 versus “patient-pre.” NS, *p* > 0.05. ROI, region of interest; L, left; R, right; MFG, middle frontal gyrus; SMA, supplementary motor area; SFG, superior frontal gyrus; MI, primary motor cortex; CMA, cingulate motor area; SI, primary somatosensory cortex.

Then, the FC between ROIs (left MFG, MI, and right CMA) (Figure S1) and their overlapped regions were selected (Table 1). To investigate the changes of the selected FC when patients recovered, we computed their FC strength for all groups, respectively (Figure 4). Consistent with our previous discovery, significantly increased FC of three ROIs was observed in the patient group. After acupuncture, all the increased FC was significantly reduced, which also happened when patients recovered (Figure S6). In addition to a pair of FC between L MFG and R SFG (A4), there is no significant FC strength difference between healthy and recovered group (Figure 4).

Finally, in order to examine whether FC modulation by acupuncture was specific in the patient group, above-mentioned three seed points were chosen for further FC analysis. By comparing the FC between post- and preacupuncture in the healthy group, no significant changes in three ROIs were observed. By comparing the FC between post- and preacupuncture in the recovered group, no significant changes in three ROIs were observed. In the meantime, by comparing the FC between the recovered group and the healthy group in the resting-state data before acupuncture, no significant changes in three ROIs were observed.

## 4. Discussion

This study aimed to investigate FC modulation induced by acupuncture in patients with Bell's palsy, which might be helpful in exploring the mechanism of acupuncture treatment. With this objective, we first selected the abnormal increased FC that could be reduced by acupuncture in the patient group (Figures 1 [Fig fig2]–3) and then investigated the FC strength of the selected FC changes with different stages. Most of the variation trends of the selected FC strength were roughly the same (Figure 4). The strength of FC reflected the degree of neuronal activity synchronization [25, 26] and also the strength of information transfer and collaboration between brain areas [23]. In this part, we discuss the selected ROIs firstly. Then, we focus on the regions in the selected FC and discuss the possible mechanism of the FC changes in patients during rehabilitation and acupuncture-induced changes in FC. Finally, we attempt to explain what these FC changes by acupuncture might imply in patients with BP.

### 4.1. Three Selected Regions of Interest

In this study, in order to examine the possible effect of acupuncture on the altered FC in patients with BP, 17 ROIs were extracted from acupuncture needling response (Table S1) and BP patients' motor task (Table S2). Many fMRI investigations about BP selected ROIs and explored the pathology in brain activation in BP patients while they were subjected to facial motor task of mouth [19–22]. Our previous study also extracted the ROIs from the acupuncture needling response to explore acupuncture-induced FC changes [18]. Thus, although these 17 seed points were from two different tasks, they were probably associated with the FC modulation by acupuncture in BP patients.

The overlapped regions were found only from three seed points (Figures 1 [Fig fig2]–3). MI and CMA were selected as the ROIs from patients' mouth motor task (Figures S1B and S1C). The MI, located in the precentral gyrus, works in association with other related motor areas to plan and execute movements. There is a broadly somatotopic representation of different body parts in the primary motor cortex in an arrangement called a motor homunculus [27]. The CMA, which is buried in the cingulate sulcus and does not extend into the cingulate gyrus [28], participates in motor control by facilitating the execution of appropriate responses and/or suppressing the execution of inappropriate ones [29]. Many previous neuroimaging studies have shown that MI and CMA could be activated in the mouth movement of patients with BP [19, 20, 22]. In the meantime, the MI in this study corresponded to the somatotopic representation of the facial MI on the contralateral side of the facial palsy and was known to display the altered FC in patients with BP [23].

MFG was selected as one of the seed regions from the activation map of the acupuncture task in the patient group (Figure S1A). Other studies on acupoint-specific fMRI patterns showed that acupuncture at LI4 produced deactivation in frontal areas [30], which were consistent with our results. MFG belonged to the dorsolateral prefrontal cortex (DLPFC). The DLPFC is directly interconnected with the sensorimotor cortex and indirectly connected with limbic structures that process internal information and is critical for arbitrary associations between sensory cues, rewards, and voluntary actions [31, 32]. Other studies concerning acupuncture treatment for pain and diarrhea demonstrated that acupuncture could change the FC of DLPFC [33, 34]. Although this seed point was not extracted from the mouth movement task, it was closely linked with the traditional sensorimotor regions [35]. In the patient, the abnormal FC that could be changed by acupuncture was found from these three ROIs probably because of the important role of these ROIs in BP.

### 4.2. Changes in Functional Connectivity with Patients during Rehabilitation

Compared with the healthy group, the increased FC was found in the patient group (Figures S1–S3A). When the patients recovered, most of the selected FC was reduced to the levels that were not significantly different from those in the healthy group (Figure 4). In order to explore the mechanism of the changes, we first identified regions in the increased FC. In this paper, significant increased FC was found in the MI, CMA, and SMA (Table 1, Figure 4). In our study, MI and CMA were selected as the seed regions located on facial motor areas. Previous investigations have reported that oral motor neurons received purely contralateral input from the cerebral cortex [36]. Other previous studies showed that FC of contralateral MI was altered in patients with BP [19, 20, 23]; our results supported their finding. The SMA is a part of the primate cerebral cortex that contributes to the control of movement. Many studies hypothesized that SMA had many functions including initiation of internally generated movement as opposed to stimulus driven movement [37] and coordinating temporal sequences of actions [38]. Both CMA and SMA are major contributors to early stage premovement activity and play an important role in the preparation and readiness for voluntary movement [39]. Therefore, considering that their FC had changed in patients with BP [19], such changes supported our results.

All of the above regions are components of the facial motor network [23, 40]. As is well known, the deafferentation (without afferent) is the most important characteristic of BP to account for the consequent impaired facial motor function of the affected side of the face. However, human brain is a complex integrative network of functionally linked brain regions, where multiple spatially distributed but functionally linked brain regions continuously share information with each other, together forming interconnected resting-state communities [41]. At the cortical level, a complex network of specialized motor areas supports voluntary facial movements [42], along with other areas especially the sensory areas playing an important role. Therefore, in healthy individuals, a balanced state is formed between the facial motor network and some other related regions, maintaining such synergies by ensuring that the facial muscles are working properly [18]. However, in patients with BP, impaired motor function initially leads to a disrupted connectivity within the cortical facial motor network and thus breaks the balance. The acute reduction of movement feedback leads to the cerebral cortical reorganization [19, 23], eventually accounting for the results that the FC between the motor association areas and other regions are changed in patients with BP. After the recovery from facial nerve palsy which is completed by cortical reorganization [23], the balance gets restored and may therefore be the reason for the increased FC changing back to a normal status. In the recovery group before acupuncture, only one pair of FC whose FC strength was different from the healthy group was found. We speculated that the reason might be that this pair of FC was not entirely restored. However, compared with the patient group, it was still reduced after the recovery.

### 4.3. Acupuncture-Induced Changes in Functional Connectivity with Patients

Compared with the preacupuncture, significant decreased FC was observed in sensorimotor related brain areas including MFG (BA9), SFG (BA10), SI, and SMA (Table 1, Figure 4). The SI is the main sensory receptive area for the sense of touch. In the neural network of facial movement, the motor command is transmitted from MI and its executive condition is fed back to SI. Previous studies of acupuncture demonstrated that the SI was activated in response to acupuncture at LI4 [43, 44]. Our previous studies have also shown that changes in FC of SI induced by acupuncture varied with onset duration of BP [18]. The other three areas were MFG (BA9), SFG (BA10), and SMA. BA9 is involved in verbal fluency [45], error detection [46], and auditory verbal attention [47]. BA10 is activated by tasks that require integration of multiple relations [48]. And it is involved in strategic processes in memory recall and various executive functions [49]. There were also many previous researches reporting that sensorimotor association areas could be affected by acupuncture [30, 34, 50, 51].

A significant decreased FC was observed in bilateral precuneus (Figures 2 and S4). The central role of this region is a wide spectrum of highly integrated tasks, including visuospatial imagery, episodic memory retrieval, and self-processing operations [52]. In Klingner et al.'s study [19], the stronger activation was observed in the precuneus during the movement of the paretic compared to those of the nonparetic side in the acute stage of BP. It demonstrated that the inability to perform the motor task led to the attempt to reactivate the image of the intended movement to support its execution; it might interpret our results in some way. In the meantime, the precuneus is known to be in the brain's default mode network (DMN), a set of areas that are spontaneously active during passive moments [53]. Recent investigations on DMN in patients demonstrated that the DMN could affect homeostasis regulation [54–56]. A lot of previous neuroimaging studies suggested that acupuncture stimulation could modulate the FC in the limbic system and DMN [34, 43, 44, 57, 58], and our results were consistent with these findings.

### 4.4. The Influence of Functional Connectivity Modulation Induced by Acupuncture

In patients with BP, we found the trend of FC changes induced by acupuncture was consistent with the changes induced by rehabilitation. As discussed above, impaired motor function might increase the FC between the motor association areas and other regions in patients. Thereafter, acupuncture could modulate the regions in the sensorimotor areas and the homeostatic related network to recover the increased FC. In the meantime, acupuncture-induced changes were only observed in the patient group, which demonstrated that acupuncture could specifically modulate the selected FC in patients with BP. This result was in accordance with our previous researches on effect of acupuncture on FC of ACC and SI for BP patients with different clinical duration [18, 59]. To some extent, our results supported the traditional Chinese medical theory that acupuncture could modulate homeostatic state of body [60]. In the end, importantly, all the selected FC returned to a normal state after the recovery, and all the patients only received acupuncture treatment in the course of rehabilitation. To account the sustained effect of acupuncture [51, 61], this demonstrated that FC modulation induced by acupuncture in patients with BP would possibly be beneficial to recovery from the disease.

BP is known as an acute idiopathic onset and self-limiting facial palsy [62]. Previous study suggests the recovery from facial nerve palsy is complemented by cortical reorganization [23]. The brain has an intrinsic capability to compensate for brain damage through reorganizing surviving networks [63]. There are many researches about cortical plasticity showing a functional plasticity in the brain of facial palsy patients in response to mere peripheral deafferentation [22, 64]. In fact, cortical reorganization is accompanied by changes of FC [21]. Our results showed that the abnormal FC could also be altered when BP patients recovered (Figures 4 and S6). Therefore, FC plays an important role in the rehabilitation of BP [19, 23]. To some extent, the above-mentioned conclusions could support our speculation that the regulation of acupuncture on the selected abnormal FC in BP patients was associated with rehabilitation.

In our previous studies on exploring the mechanism of acupuncture treatment for BP, we found that FC could be changed by acupuncture in BP patients [18, 59]. However, these preliminary findings cannot determine whether the effect of acupuncture on FC was associated with rehabilitation. In this paper, through a more in-depth research about FC modulation of acupuncture, we discovered that acupuncture could regulate some abnormal FC in BP patients and proved that the effort of acupuncture is consistent with the tendency of recovery.

## 5. Limitations and Conclusion

Although important discoveries were made by present studies, there were also limitations. First, the ROIs in this study were selected based on the activation mapping in the mouth movement task and acupuncture task sessions. Thus, only the regions where alterations were reduced by acupuncture were selected; although other ROIs and the altered FC might explain some of the problems about BP, they were ignored. Second, in this study, there was no sham acupuncture control group due to ethics restriction. Thus, the changes observed in this study may not be necessarily specific to acupuncture, which may be similarly observed in other somatic stimulations. Finally, a single acupoint (Hegu) was stimulated in the scanning process. In fact, when the patients were treated with acupuncture, multiple acupoints were stimulated at the same time. In the future, we may design the task to stimulate multiple acupoints to explore the mechanism of acupuncture for BP. In terms of these limitations, further investigations with stricter control of these relevant variables should be done to provide more convincing evidences to support our conclusion.

In summary, the present study suggests that acupuncture can specifically reduce some FC that may be increased by BP. Then, the results reflect that FC modulation induced by acupuncture in patients with BP is consistent with the tendency of recovery, which may be beneficial to recovery from the disease. Although further researches are still needed, the finding hopes to shed light on the clarification of the underlying mechanism of acupuncture on LI4 for BP.

## Supplementary Material

Many details about the methods and results are provided in the Supplementary Material, including more details about the seed points (Figure S1, Table S1 and S2), the acupuncture stimulation task (Figure S2) and the intergroup comparison results (Figure S3-S6, Table S3 and S4).

## Figures and Tables

**Table 1:** All the overlapped regions were selected from two intergroup comparison functional connectivity results.

ROI	Common regions (BA)	Side	MNI coordinates			Voxel	Number
			*X* (mm)	*Y* (mm)	*Z* (mm)		
L MFG	Supplementary motor area	R	4	8	52	66	A1
	Supplementary motor area	L	−1	−23	53	66	A2
	Superior frontal gyrus (10)	R	32	50	31	48	A3
	Superior frontal gyrus	R	17	35	45	27	A4

L MI	Middle frontal gyrus (9)	L	−48	14	32	78	B1
	Precuneus	R	6	−67	53	76	B2
	Middle frontal gyrus	L	−35	19	26	32	B3
	Precuneus	L	−1	−62	39	29	B4
	Middle frontal gyrus	L	−37	9	52	20	B5

R CMA	Supplementary motor area	R	3	−12	54	123	C1
	Postcentral gyrus, SI	R	3	−49	67	82	C2

Note: ROI, region of interest; BA, Brodmann area; MFG, middle frontal gyrus; CMA, cingulate motor area; MI, primary motor cortex; SI, primary somatosensory cortex; R, right; L, left. The overlapped regions of less than 15 voxels were ignored. Coordinate point is the geometrical center of the overlapped region.
