# Supplementary material for: Functional Connectivity Modulation by Acupuncture in Patients with Bell's Palsy
Source: Evid Based Complement Alternat Med. 2016 May 12;2016:5928758. doi: 10.1155/2016/5928758 (PMC4880700; doi:10.1155/2016/5928758)
Supplement: Supplementary file 1 — Many details about the methods and results are provided in the Supplementary Material, including more details about the seed points (Figure S1, Table S1 and S2), the acupuncture stimulation task (Figure S2) and the intergroup comparison results (Figure S3-S6, Table S3 and S4). [file 5928758.f1.pdf]

Supplementary materials for

Functional connectivity modulation by acupuncture in patients with Bell's palsy

Yunpeng Bian, Xiaoxuan He, Sheng Hu, Chuanfu Li, Chunsheng Xu, Hongxing Kan, Qiuju Xue, Jun Yang, and Bensheng Qiu

This PDF file includes

Fig.S1-S6
Table S1-S4

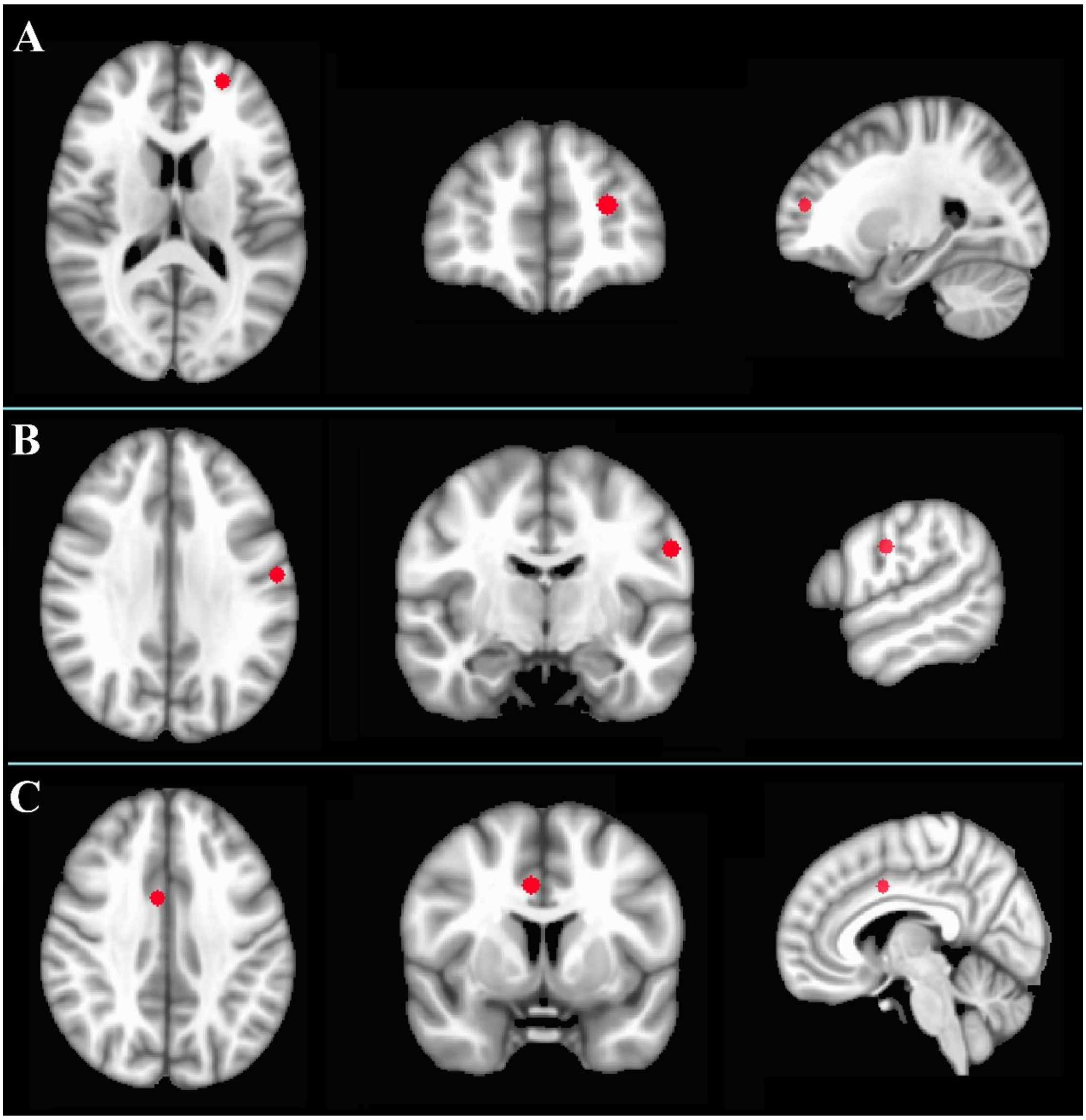

Figure S1: Demonstration of the selected ROI of the left middle frontal gyrus (A), left primary motor cortex (B), right cingulate motor area (C). ROI, region of interest.

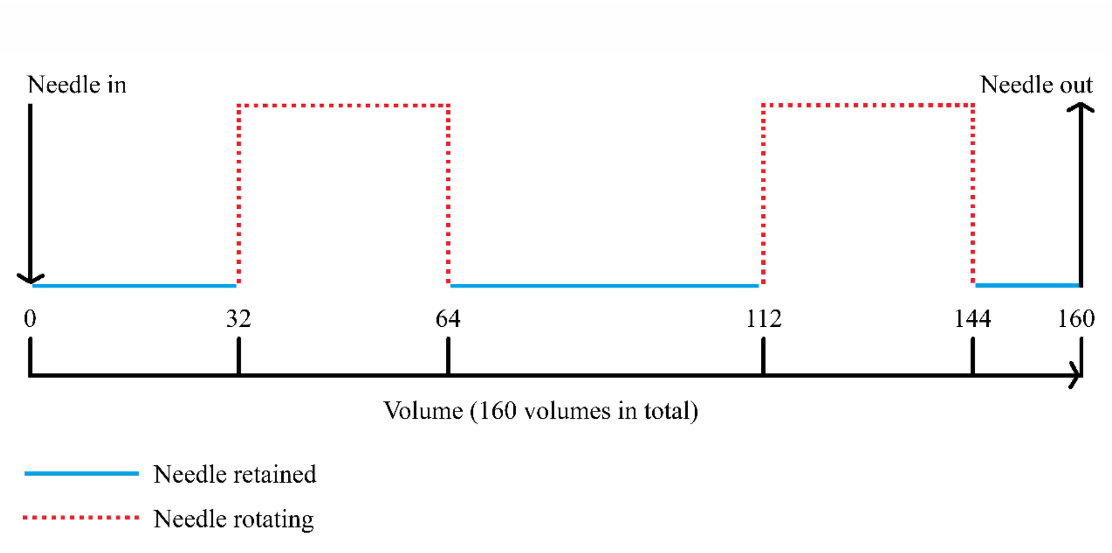

Figure S2: Diagram for the acupuncture stimulation task. The needle was retained in the skin after de-qi, and the acquisition began. After remaining at rest for 32 volumes, the needle was rotated bidirectionally with an even motion at the rate of 1Hz for 32 volumes. After another rest period of 48 volumes, needle manipulation was repeated in a similar manner for another 32 volumes. After that, scanning continued for 16 volumes, and the needle was pulled out.

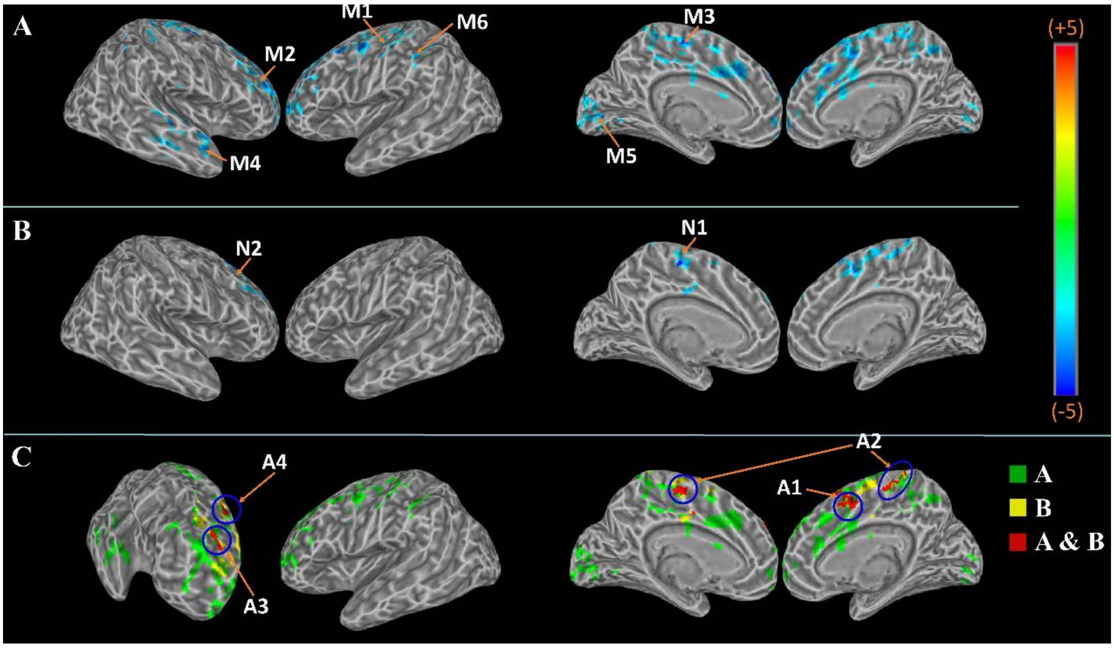

Figure S3: Demonstration of the intergroup comparison functional connectivity (FC) results of left middle frontal gyrus. The threshold was set to $P \leq 0.05$, $\alpha \leq 0.05$, corrected using the Monte Carlo method. (A) Intergroup FC results of areas between the healthy group and the patient group before acupuncture. A significant decrease in left middle frontal gyrus connectivity was observed in the left middle frontal gyrus (M1), medial frontal gyrus (M3), cuneus (M5), postcentral gyrus (M6) and right superior frontal gyrus (M2), middle temporal gyrus (M4). (B) Intergroup FC results of areas between post-and pre-acupuncture in the patient group. A significant decrease in left middle frontal gyrus connectivity was observed in the left medial frontal gyrus (N1) and right superior frontal gyrus. (C) Comparison of the

results of A and B, the overlapped regions were detected in the bilateral supplementary motor area (A1, A2) and right superior frontal gyrus (A3, A4).

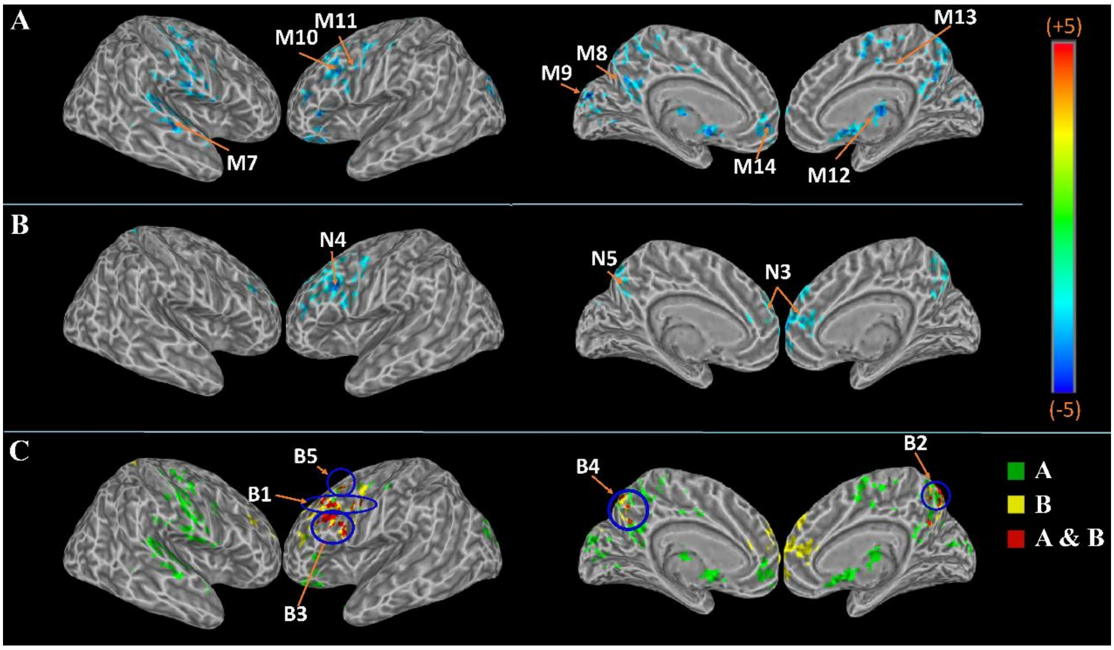

Figure S4: Demonstration of the intergroup comparison functional connectivity (FC) results of left primary motor cortex. The threshold was set to $P \leq 0.05$, $\alpha \leq 0.05$, corrected using the Monte Carlo method. (A) Intergroup FC results of areas between the healthy group and the patient group before acupuncture. A significant decrease in left primary motor cortex connectivity was observed in the left precuneus (M8), cuneus (M9), middle frontal gyrus (M10), inferior frontal gyrus (M11), medial frontal gyrus (M14) and right superior temporal gyrus (M7), thalamus (M12), paracentral lobule (M13). (B) Intergroup FC results of areas between post-and pre-acupuncture in the patient group. A significant decrease in left primary motor cortex connectivity was observed in the left superior frontal gyrus (N3), middle frontal gyrus (N4) and precuneus (N5). (C) Comparison of the results of A and B, the overlapped regions were detected in the left middle frontal gyrus (B1, B3, B5), left precuneus (B4) and right precuneus (B2).

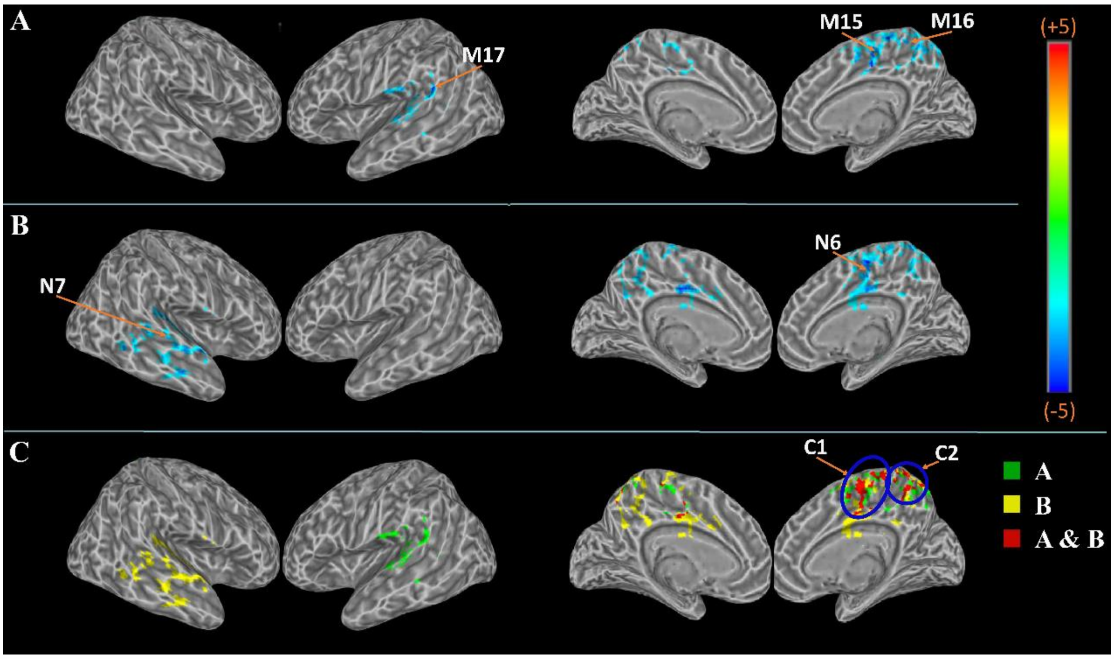

Figure S5: Demonstration of the intergroup comparison functional connectivity (FC) results of right cingulate motor area. The threshold was set to $P \leq 0.05$, $\alpha \leq 0.05$, corrected using the Monte Carlo method. (A) Intergroup comparison FC results of areas between the healthy group and the patient group before acupuncture. A significant decrease in right cingulate motor area connectivity was observed in the right cingulate gyrus (M15), paracentral louble (M16) and left superior temporal gyrus (M17). (B) Intergroup FC results of areas between post-and pre-acupuncture in the patient group. A significant decrease in right cingulate motor area connectivity was observed in the right cingulate gyrus (N6) and superior temporal gyrus (N7). (C) Comparison of the results of A and B, the overlapped regions were detected in the right supplementary motor area (C1) and right primary somatosensory cortex (C2).

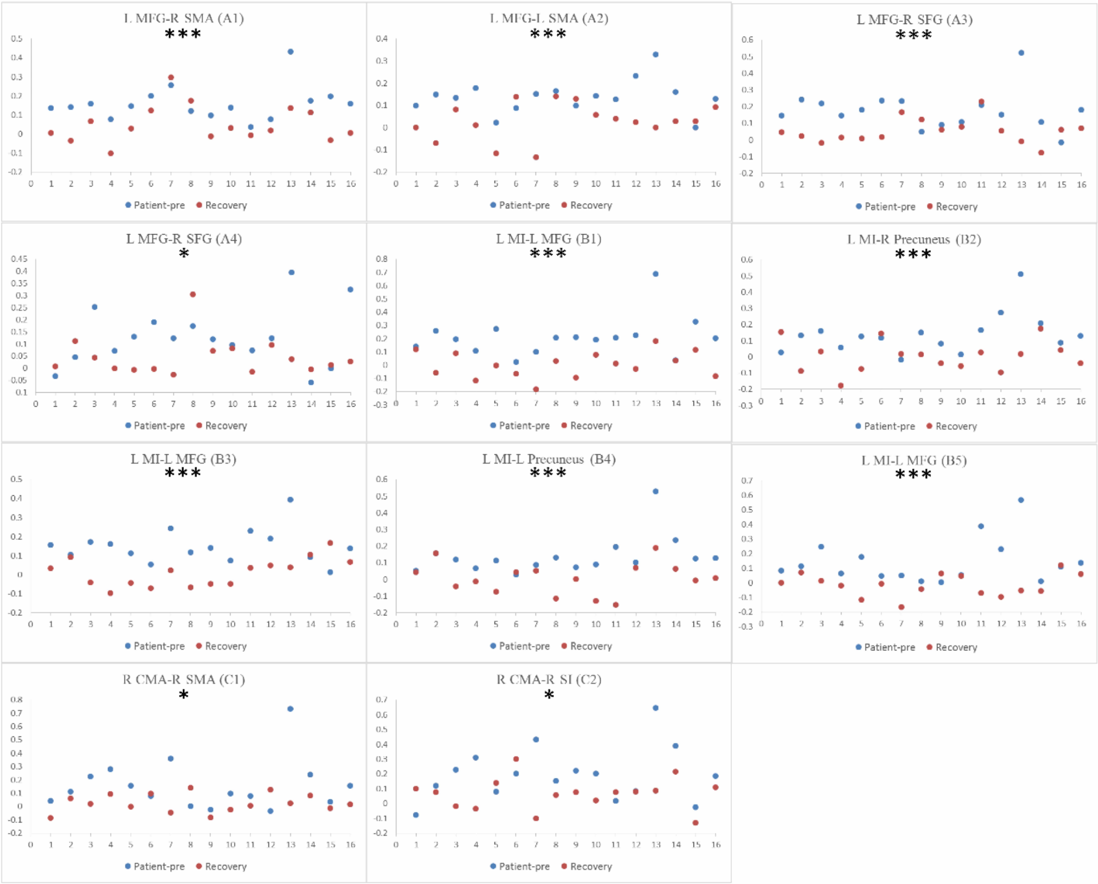

Figure S6: Scatter plot of functional connectivity (FC) strength of each individual patient in the patient group and the recovery group. Reduction of these selected FC was observed when the patients recovered. The x-axis represents different patient (due to loss of contact, one female did not receive scanning after the recovery), and the y-axis represents the FC strength. 'Patient-pre' represents the patient group before acupuncture, 'Recovery' represents the recovered group before acupuncture. The intergroup comparisons were performed by paired t-test, *** $p < 0.005$, * $p < 0.05$. L, left; R, right; MFG, middle frontal gyrus; SMA, supplementary motor area; SFG, superior frontal gyrus; MI, primary motor cortex; CMA, cingulate motor area; SI, primary somatosensory cortex.

Table S1: Group analysis results of areas in the task-state fMRI data of the patient group

Regions(BA)	Side	MNI coordinates			Peak(*Z*-value)
		X(mm)	*Y*(mm)	*Z*(mm)	
Precentral gyrus (6)	L	-39	-9	30	-3.160
Middle frontal gyrus	L	-24	51	15	-4.174
Middle frontal gyrus (9)	L	-30	36	33	-3.710
Postcentral gyrus (2)	L	-51	-30	42	-3.906
Inferior frontal gyrus (44)	L	-60	15	18	-3.796
Cingulate gyrus	R	12	15	45	-3.665
Inferior parietal lobule	L	-30	-48	42	-3.478

Note: BA, Brodmann area; R, right; L, left. The threshold was set to $P \leq 0.01$, $\alpha \leq 0.05$ (corrected using the Monte Carlo method).

Table S2: The seed regions were selected in Klingner's study

Regions(BA)	Side	MNI coordinates		
		X(mm)	*Y*(mm)	*Z*(mm)
Primary motor cortex, MI	L	-57	-10	31
Primary motor cortex, MI	R	51	-10	34
Cerebellum	L	-42	-52	-29
Cerebellum	R	33	-55	-29
Ventral lateral premotor cortex	L	-57	-1	25
Ventral lateral premotor cortex	R	63	-1	19
Supplementary motor area	L	-3	2	55
Supplementary motor area	R	3	5	55
Cingulate motor area	L	-3	5	40
Cingulate motor area	R	6	5	37

Note: BA, Brodmann area; MI, primary motor cortex; R, right; L, left.

Table S3: Intergroup functional connectivity analysis results of areas between the healthy group and the patient group before acupuncture

ROI	Regions (BA)	Side	MNI coordinates			Peak(*Z*-value)	Number
			X(mm)	*Y*(mm)	*Z*(mm)		
L MFG	Middle Frontal Gyrus	L	-33	-12	45	-4.253	M1
	Superior Frontal Gyrus (9)	R	33	45	33	-4.187	M2
	Medial Frontal Gyrus	L	0	-21	51	-3.856	M3
	Middle Temporal Gyrus (21)	R	60	3	-12	-3.492	M4
	Cuneus (18)	L	-3	-81	3	-4.059	M5
	Postcentral Gyrus (40)	L	-54	-33	51	-3.665	M6
L MI	Superior Temporal Gyrus	R	54	-18	0	-3.709	M7
	Precuneus	L	0	-60	36	-3.803	M8
	Cuneus	L	-6	-84	21	-4.101	M9
	Middle Frontal Gyrus	L	-36	18	27	-4.073	M10
	Inferior Frontal Gyrus	L	-48	6	30	-3.563	M11
	Thalamus	R	6	-9	0	-4.294	M12
	Paracentral Louble (31)	R	3	-24	48	-3.959	M13
	Medial Frontal Gyrus	L	-15	51	-6	-3.500	M14
R CMA	Cingulate Gyrus (24)	R	3	-6	42	-4.166	M15
	Paracentral Louble (5)	R	3	-45	57	-3.395	M16
	Superior Temporal Gyrus(22)	L	-63	-45	18	-3.997	M17

Note: ROI, region of interest; BA, Brodmann area; MFG, middle frontal gyrus; CMA, cingulate motor area; MI, primary motor cortex; R, right; L, left. The threshold was set to $P \leq 0.05$, $\alpha \leq 0.05$ (corrected using the Monte Carlo method).

Table S4: Intergroup functional connectivity analysis results of areas between post-and pre-acupuncture in the patient group.

ROI	Common Regions (BA)	Side	MNI coordinates			Peak(*Z*-value)	Number
			X(mm)	*Y*(mm)	*Z*(mm)		
L MFG	Medial Frontal Gyrus	L	-3	-21	51	-4.191	N1
	Superior Frontal Gyrus	R	18	30	48	-3.534	N2
L MI	Superior Frontal Gyrus(9)	L	-12	54	39	-4.001	N3
	Middle Frontal Gyrus	L	-36	18	27	-4.354	N4
	Precuneus (7)	L	-3	-69	45	-3.094	N5
R CMA	Cingulate Gyrus (24)	R	6	-9	47	-4.149	N6
	Superior Temporal Gyrus	R	48	-30	3	-4.177	N7

Note: ROI, region of interest; BA, Brodmann area; MFG, middle frontal gyrus; CMA, cingulate motor area; MI, primary motor cortex; R, right; L, left. The threshold was set to $P \leq 0.05$, $\alpha \leq 0.05$ (corrected using the Monte Carlo method).
